# Specimen adequacy assay controls in nucleic acid amplification tests do not correlate with nasopharyngeal swab collection method

**DOI:** 10.1128/jcm.00975-24

**Published:** 2024-09-16

**Authors:** Katharine H. D. Crawford, Mary Lynn Baniecki, Elizabeth G. Dushin, Cassandra A. Tierney, Shunjie Guan, Laurence L. Stensland, Ailyn C. Perez-Osorio, Alexander L. Greninger

**Affiliations:** 1 Department of Laboratory Medicine and Pathology, University of Washington, Seattle, Washington, USA; 2 Pfizer, Inc., Cambridge, Massachusetts, USA; 3 Pfizer, Inc., Groton, Connecticut, USA; 4 Vaccine and Infectious Disease Division, Fred Hutchinson Research Center, Seattle, Washington, USA; St. Jude Children's Research Hospital, Memphis, Tennessee, USA

**Keywords:** internal control, housekeeping gene, specimen adequacy, control, nasopharyngeal swab, swab

## LETTER

Nasopharyngeal swabs are commonly used to test for respiratory virus infections but require trained personnel for proper collection (1–7). During the COVID-19 pandemic, nasal swabs were frequently self-collected, increasing access to testing, but decreasing true sampling of the nasopharynx and likely increasing variability in collection quality (8, 9). Throughout clinical microbiology, the increased use of self-collected specimens has driven interest in the use of internal controls to monitor specimen collection adequacy in nucleic acid amplification tests (10, 11). However, there are limited data on the utility and interpretation of these controls, especially from prospective clinical studies (12–14). To better understand the impact of swab collection quality on specimen adequacy internal control results, we conducted a clinical study to examine qPCR results for two commonly used human housekeeping genes—ribonuclease P protein subunit p30 (RPP30) and β-globin—from nasopharyngeal swabs collected under pre-specified collection conditions.

iSwab-plus flocked nasopharyngeal swabs were collected by trained personnel entering both nares or a single naris with good, suboptimal, or poor collection quality defined by the distance and time of insertion in the participants’ nares. “Good” quality swabs were inserted fully into the nasopharynx and rotated at least 5 seconds per naris. “Suboptimal” quality nasopharyngeal swabs were inserted into the nasopharynx, but only long enough to complete one rotation of the swab (generally <3 seconds). “Poor” quality swabs were inserted into the nostril only and promptly removed without swab rotation and essentially constituted an anterior nasal collection using a nasopharyngeal swab. Dry nasopharyngeal swabs were placed in transport tubes and frozen at ≤−70°C prior to elution in 3 mL RPMI + 2% FBS viral transport medium in the laboratory (15).

We used qPCR amplification of the genes encoding RPP30 or β-globin to measure the impact of nasopharyngeal swab collection quality on nucleic acid recovery. Samples underwent nucleic acid extraction and qPCR in the University of Washington Virology laboratory (Supplemental Methods). The RPP30 assay includes a reverse transcription step and detects both mRNA and DNA, resulting in a slightly greater detection rate and lower cycle threshold (Ct) values compared to the β-globin assay, which only detects DNA.

**Fig 1:**
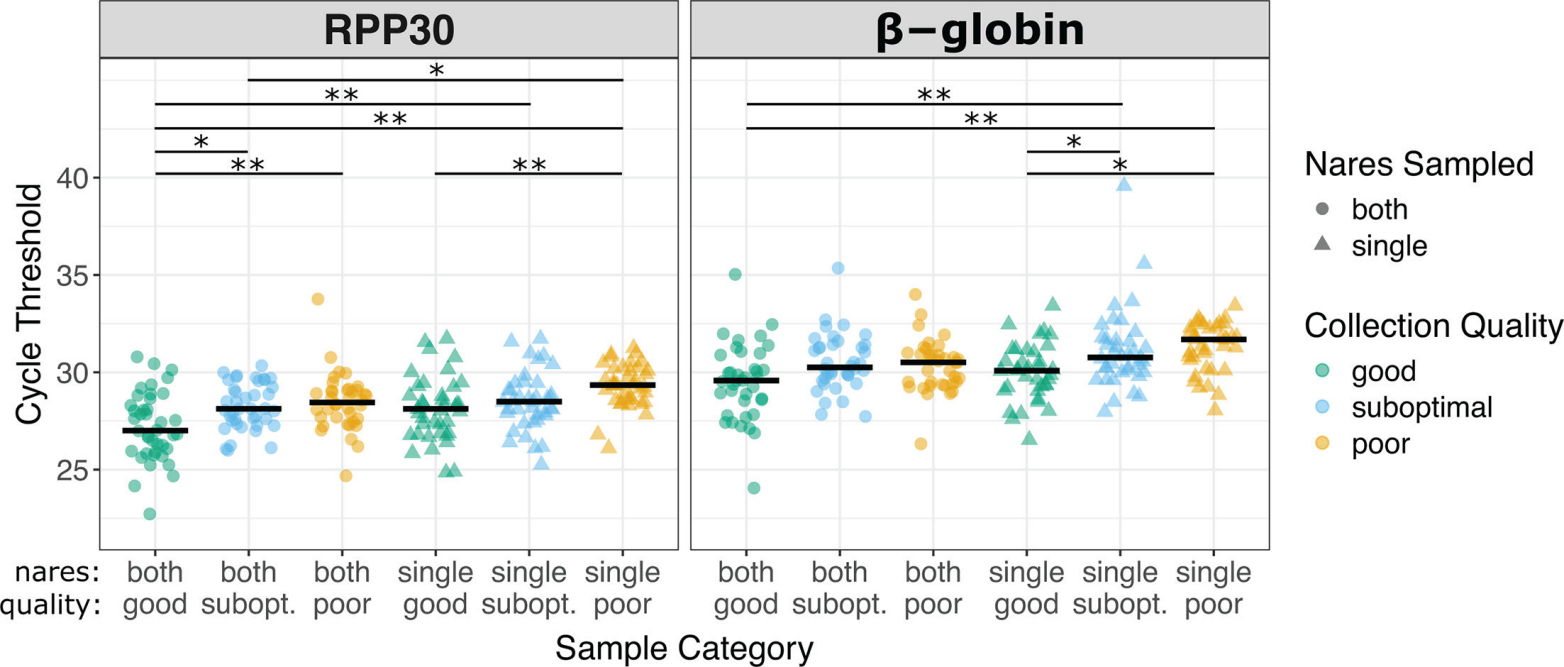
Cycle threshold (Ct) values for the RPP30 and β-globin qPCR assays. “Good” nasopharyngeal swabs were collected from the nasopharynx with rotation for at least 5 seconds per naris, while “suboptimal” swabs were similarly inserted but for only one rotation of the swab (<3 seconds). “Poor” quality swabs did not fully reach the nasopharyngeal cavity and included no swab rotation. Significant differences in Ct value are shown on the plot (ANOVA test, *P < 0.05 and **P < 0.01). The horizontal black line indicates median Ct values. The samples whose RPP30 or β-globin genes did not amplify (see Table 1) are not included in this analysis. Suboptimal is abbreviated to “subopt.” in the *x*-axis labels for space considerations.

Forty swabs were collected for each collection condition and tested for both housekeeping genes. RPP30 was detected in ≥95% and β-globin in ≥90% of samples from each condition (Table 1). There was no significant difference in the ability to detect RPP30 or β-globin based on the sample collection method (Chi-squared test, RPP30: *P* = 0.63; β-globin: *P* = 0.93). Excluding samples without detection of the tested housekeeping gene, samples collected with a “good” collection method on average had significantly lower Ct values than samples with a poor collection method (Fig. 1). However, there was substantial overlap in the Ct value distribution preventing definitive assignment of collection method by Ct value.

**TABLE 1:** Number of samples with detectable RPP30 or β-globin by qPCR

		RPP30		β-globin	
Nares	Collection quality	Number detected	% Detected	Number detected	% Detected
Both	Good	40/40	100	38/40	95
	Suboptimal	40/40	100	38/40	95
	Poor	38/40	95	37/40	93
Single	Good	39/40	98	36/40	90
	Suboptimal	39/40	98	38/40	95
	Poor	39/40	98	37/40	93

Overall, our data show that quantitative specimen adequacy control qPCR results are not fully representative of the nasopharyngeal swab collection method. A concerted strength of this study was to isolate the collection method as a variable for prospective clinical study, rather than *post hoc* analyses of specimens collected for viral testing. Limitations include the use of specimen adequacy control assays that can detect DNA—though these two assays are commonly used for specimen adequacy (especially RPP30, which was included in CDC COVID-19 assay (16)—as well as the interrogation of nasopharyngeal swabs which are not generally self-collected). However, using nasopharyngeal swabs allowed a wider interrogation of specimen collection method/quality by trained staff, making the significant overlap in housekeeping Ct values even more notable. Overall, these data highlight the limitations of interpreting quantitative specimen adequacy controls and the need to conduct prospective clinical studies to interrogate them (17, 18).

## SUPPLEMENTAL MATERIAL

Supplemental material jcm.00975-24-s0001.docx Supplemental methods; Tables S1 and S2.

ASM does not own the copyrights to Supplemental Material that may be linked to, or accessed through, an article. The authors have granted ASM a non-exclusive, world-wide license to publish the Supplemental Material files. Please contact the corresponding author directly for reuse.
