# Supplementary material for: Specimen adequacy assay controls in nucleic acid amplification tests do not correlate with nasopharyngeal swab collection method
Source: J Clin Microbiol. 2024 Sep 16;62(10):e00975-24. doi: 10.1128/jcm.00975-24 (PMC11481563; doi:10.1128/jcm.00975-24)
Supplement: Supplemental material — Supplemental methods; Tables S1 and S2. [file jcm.00975-24-s0001.docx]

**Supplemental Methods**

Samples were collected from 20 different participants on two separate visits one week apart. Each participant contributed one iSwab-plus (KFDA registration number 4656) nasopharyngeal swab for each sample collection category (6 swabs total) at each time point resulting in 40 swabs per collection method (“good”, “suboptimal”, and “poor”) and 240 swabs total. A secondary analysis of housekeeping gene expression by sample collection quality on each date (excluding samples with undetectable RPP30 or β-globin) found that only the single naris, suboptimal collection group had a significant difference in Ct value by date (paired t-test, p-value = 0.048), presumably due to a clear outlier sample with a high, but detectable Ct value. For our analyses, each NP swab was treated independently.

After collection, NP swabs were stored dry in 15mL Falcon tube at ≤ -70 °C until use in this study. After receipt in the University of Washington Virology laboratory, swabs were resuspended in 3 mL of RPMI + 2% fetal bovine serum, vortexed for 3 seconds and incubated at room temperature for 15 minutes. RPP30 specificity testing using RPMI+FBS or NP swabs dipped in RPMI+FBS showed no reactivity. Total nucleic acid (TNA) was then extracted from 200 µL of each sample using the MagnaPure 96 Small Volume nucleic acid extraction kit (Roche, Indianapolis, IN) and eluted into 50 µL buffer.

For RPP30, testing was performed using Taqman real-time reverse transcription qPCR on an Applied Biosystems QuantStudio 7 instrument with primers and probe targeting a 62 bp fragment of the RPP30 gene (Supplemental Table 1). Each reaction was performed using 10 µL of extracted TNA, 6 µL 5x QuantiTect Virus Master Mix, 1.5 µL 20x primer + probe master mix (final concentrations: 0.9 µM of each primer and 0.25 µM of the probe), 0.3 µL QuantiTect Virus RT mix, 0.6 µL 50x ROX Dye Solution, and 11.6 µL water. Cycling conditions were: 50 °C 20 min, 95 °C 5 min, and 45 cycles of: 94 °C 15 sec, 60 °C 45 sec. The RPP30 assay has a limit of detection at a threshold of 0.2 of a Ct value of 38.

The β-globin assay is similarly performed using real-time qPCR on an Applied Biosystems QuantStuido 7 instrument. However, the β-globin assay does not include a reverse transcription step, and thereby only detects DNA. The β-globin primers and probe target a 72 bp fragment of the β-globin gene (Supplemental Table 1). Each reaction was performed using: 10 µL extracted total nucleic acid, 14.33 µL 2x QuantiTect Multiplex PCR NoROX Master Mix, 0.67 µL 2x QuantiTect Multiplex PCR Master Mix (with ROX), 0.10 µL EXO internal control mix (master mix recipe in Supplemental Table 2), 0.075 µL forward primer (0.83 µM final concentration), 0.075 µL reverse primer (0.83 µM final concentration), 0.03 µL probe (0.1 µM final concentration), 0.03 µL UNG, and 4.69 µL water. Cycling conditions were: 50 °C 2 min, 95 °C 15 min, and 45 cycles of: 94 °C 1 min, 60 °C 1 min. The β-globin assay includes a gDNA standard curve to allow for absolute quantitation of β-globin copies/reaction. The limit of detection with a threshold of 0.5 is 2.69 copies/reaction for clinical NP swab remnant samples. However, for our analyses, we only included the β-globin Ct values for any detectable sample—not the copies/reaction quantitative results—to allow for a more direct comparison with the RPP30 assay.

Data analysis was performed using R with statistical tests described where relevant.

**Supplemental Table 1:** Primer and Probe Sequences

| **Primer** | **Sequence** |
| --- | --- |
| RPP30 F | GAT TTG GAC CTG CGA GCG |
| RPP30 R | GCG GCT GTC TCC ACA AGT |
| RPP30 P | VIC-TCT GAC CTG AAG GCT CTG CGC G–TAMRA |
| BETA F | TGA AGG CTC ATG GCA AGA AA |
| BETA R | GCT CAC TCA GTG TGG CAA AGG |
| BETA P | FAM-TCC AGG TGA GCC AGG CCA TCA CTA-TAMRA |
| EXO-186 F | GCC TGG TGC AAA AAT TGC TT |
| EXO-315 R | TCG TTC ATT TGT TCT TTT GTG GAA |
| EXO PROBE-242t | VIC- CAG CTA TTG CAA ACG CCA TCG CAC –TAMRA |

**Supplemental Table 2**: EXO Internal Control Preparation; Prepare an Exo Master Mix according to the recipe below;

| **EXO Ingredients** | | **# of 20µL Batches** | Final Concentration in 30µL PCR |
| --- | --- | --- | --- |
| Water |  | 9.4 µL |  |
| EXO Probe 100µM | | 7.5 µL | 125nM |
| EXO F Primers 400µM | | 1.5 µL | 100nM |
| EXO R Primers 400µM | | 1.5 µL | 100nM |
| EXO Template 5e8/µL | | 0.1 µL | 8000 copies |
| **Total Mix Volume:** | | 20µl |  |

EXO Template – A plasmid named EXO containing the amplicon areas of the EXO (jellyfish DNA sequence) is purchased from Blue Heron Bio.
